# Characteristics and neural-like differentiation of mesenchymal stem cells derived from foetal porcine bone marrow

**DOI:** 10.1042/BSR20120023

**Published:** 2013-03-28

**Authors:** Ying Liu, Limei Liu, Xin Ma, Yupeng Yin, Bo Tang, Ziyi Li

**Affiliations:** *Jilin Provincial Key Laboratory of Animal Embryo Engineering, the Centre for Animal Embryo Engineering of Jilin Province, College of Animal Science and Veterinary Medicine, Jilin University, 5333 Xi An Da Lu, Changchun, Jilin 130062, China; †Department of Histology and Embryology, Norman Bethune College of Medicine, Jilin University, Changchun 130021, China; ‡Beihua University, Jilin 132021, China; §College of Animal Science and Technology, Jilin Agricultural University, Changchun 130118, China

**Keywords:** differentiation, mesenchymal stem cell, octamer-binding transcription factor 4, porcine bone marrow, reverse transcription–PCR, bFGF, basic fibroblast growth factor, COC, cumulus–oocyte complex, DMEM, Dulbecco’s modified Eagle’s medium, EGF, epidermal growth factor, ESC, embryonic stem cell, FBS, foetal bovine serum, GAPDH, glyceraldehyde-3-phosphate dehydrogenase, IVM, *in vitro* maturation, MAP2, microtubule-associated protein 2, MSC, mesenchymal stem cell, NSC, neural stem cell, Oct4, octamer-binding transcription factor 4, PB, parthenogenetic blastocyst, PI, propidium iodide, pMSC, porcine MSC, PPARG, peroxisome-proliferator-activated receptor γ, PVA, poly(vinyl alcohol), qRT-PCR, quantitative real-time PCR, RA, retinoic acid, RT–PCR, reverse transcription–PCR, TGFβ, transforming growth factor β

## Abstract

MSCs (mesenchymal stem cells) are a stem cell source that can be easily obtained from bone marrow. Despite the increasing importance of the pig as a large animal model, little is known about foetal pMSCs (porcine MSCs). In this study, we observed the gene expression of pluripotent markers in foetal pMSCs and the capacity of pMSCs to differentiate into adipocytes, osteocytes and neural-like cells using quantitative RT–PCR (reverse transcription–PCR), normal histological staining and immunohistochemistry. Foetal pMSCs have either a spindle or a flattened shape, and flow cytometry revealed the expression of the MSC-related proteins CD44 and CD105 (endoglin) but not CD34 and CD45. pMSCs express pluripotent markers such as Oct4 (octamer-binding transcription factor 4) and Nanog at the protein and mRNA levels. qRT-PCR (quantitative real-time PCR) analyses revealed that pMSCs expressed nestin [for NSCs (neural stem cells)]. Immunocytochemical and RT–PCR data showed that 29% and 23% of pMSCs expressed MAP2 (microtubule-associated protein 2) for neurons and β-tubulin III (Tuj1) for immature neurons, respectively, after induction of neural differentiation. These findings demonstrate the plasticity of pMSCs and their potential for use in cellular replacement therapy for neural diseases.

## INTRODUCTION

Compared with small animals such as rodents, large animals are superior in many aspects for the study of human diseases and for pre-clinical therapies. Pigs have been used as one of the large animal models in medical studies for scientific, economic and ethical reasons. Pigs are similar to humans in many aspects of anatomy, development, physiology, pathophysiology and disease occurrence [1,2].

MSCs (mesenchymal stem cells) are a stem cell source that can easily be obtained from bone marrow. Multipotent human MSCs express the Oct4 (octamer-binding transcription factor 4), SSEA-4 (stage-specific embryonic antigen 4) and REX-1 (RNA exonuclease homologue 1) transcription factors, which are specific markers of ESCs (embryonic stem cells) [3,4]. The potential of MSCs to form bone, cartilage and adipose tissues both *in vivo* and *in vitro* has been well documented [5]. Some reports have suggested that rat MSCs can differentiate into neurons [6]. MSCs partially originate from the neural crest during development [7]. Numerous laboratories are performing safety and efficacy studies using MSCs for the treatment of a number of neurological diseases.

The techniques used for the purification, expansion and the osteogenic, chondrogenic and adipogenic differentiation of human MSCs can be adopted for the analysis of pMSCs (porcine MSCs), which may meet the increasing demand for stem and progenitor cells in tissue engineering. pMSCs are multipotent and have the capacity for neural, adipose and osteogenic differentiation [8,9]. MSCs from large animals such as the pig offer great potential to investigate cell differentiation and cell fate. To further understand pMSC ontogeny, we isolated MSCs from foetal porcine bone marrow, observed the gene expression of pluripotent markers compared with PBs (parthenogenetic blastocysts) and determined the capacity of these cells to differentiate into neural-like cells using qRT-PCR (quantitative real-time PCR) and immunohistochemistry. As there was little information about the differentiation of pMSCs *in vitro*, the current study was conducted to investigate the differentiation of pMSCs into neurons after treatment with RA (retinoic acid) and presents a significant contribution to our understanding of the characteristics of pMSCs.

## METHODS AND MATERIALS

### Oocyte collection and IVM (*in vitro* maturation)

Porcine ovaries were collected at a local commercial abattoir and were transported to the laboratory in PBS (35–39°C) within 2 h. The ovaries were washed three times in PBS (39°C). Follicular fluid and COCs (cumulus–oocyte complexes) were aspirated from 2 to 8 mm antral follicles with a 10 ml disposable syringe and an 18 gauge needle and were expelled into sterile Petri dishes (9 cm diameter) kept at 39°C. The COCs with more than three layers of intact and compact cumulus were selected, washed three times in manipulation fluid {TCM-199 supplemented with 0.1% PVA [poly(vinyl alcohol)]} and cultured in IVM media [TCM-199 supplemented with 3.05 mM glucose, 0.91 mM sodium pyruvate, 0.57 mM cysteine, 10 ng/ml EGF (epidermal growth factor), 0.5 I.U. (international units)/ml porcine LH, 0.5 I.U./ml porcine FSH (follicle-stimulating hormone), 0.1% (w/v) PVA, 10% FBS (foetal bovine serum), 75 mg/ml penicillin and 50 mg/ml streptomycin]. A group of 10–20 oocytes was cultured in a 100 μl drop of maturation medium for up to 44 h at 39°C in an atmosphere of 5% CO_2_ and saturated humidity. All maturation media drops containing oocytes were covered with a thin layer of mineral oil and were incubated in pre-equilibrated culture medium [10].

### Electronic activation and culture of PB

After IVM, cumulus cells were removed from matured oocytes by vortex mixing for 3 min in Hepes-buffered medium with 0.1% (w/v) hyaluronidase, and the oocytes were equilibrated in activation medium [0.3 M mannitol, 0.5 mM Hepes, 0.01% (w/v) BSA, 0.1 mM CaCl_2_, 0.1 mM MgCl_2_ and 0.01% (w/v) PVA] for 5 min and placed in an activation chamber with electrodes 1 mm apart containing the activation medium. Oocytes were stimulated twice with a 30 μs pulse of 2.2 kV/mm direct current. The activated oocytes were cultured in PZM3 (porcine zygote medium 3) [11] with 5% CO_2_ in air at 39°C for 7 days. The PB derived from the activated oocytes were used as a control to compare with the pluripotency gene expression of MSCs.

### Preparation and cultivation of pMSCs

Femurs were collected from foetal pigs for pMSC isolation. The ends of the bones were cut, and the marrow was extruded with 10 ml of D-Hank's solution using a syringe. Approximately 2×10^7^ marrow cells were plated on a 100 mm diameter Petri dish in DMEM (Dulbecco's modified Eagle's medium)/F12 (Hyclone), supplemented with 10% FBS (PAA Laboratories), 2 mM L-glutamine, 100 units/ml penicillin and 100 mg/ml streptomycin. All of the cells were incubated at 37°C with 5% humidified CO_2_. After 24 h, the non-adherent cells were removed by replacing the media. The medium was replaced every 3 or 4 days for approximately 2 weeks. When the primary culture reached confluence, the cells were subcultured by trypsinization.

### Surface antigen analysis of pMSCs by flow cytometry

To determine the surface antigens of pMSCs, cells at P3 (passage 3) were treated with 0.25% trypsin and washed twice with PBS. At least 1×10^4^ cells were analysed per sample. Cells were incubated with antibodies for 30 min at 4°C and resuspended in 100 μl of PBS. Unbound antibodies were removed by washing with PBS. The cells were then analysed with a FACSCalibur™ flow cytometer (Becton Dickinson).

The antibodies included rat anti-CD44 (clone IM7), mouse anti-CD34 (clone 581) and mouse anti-CD45 (clone HI30) from BD Biosciences and mouse anti-CD105 (clone MEM 229) from Abcam, UK [12].

### Cell cycle and apoptosis assay of pMSCs

pMSCs were collected at P10 (passage 10) and fixed with ice-cold 70% ethanol overnight at −20°C. The samples were then centrifuged at 200 ***g*** for 10 min and stained with PI (propidium iodide, Beijing Dingguo), and the cell cycle data were collected by using a FACSCalibur™ flow cytometer.

Apoptosis assays were performed using the Annexin V-FITC apoptosis antibody (catalogue no. 556419; BD Pharmingen) according to the manufacturer's instructions. Briefly, cells at P10 were collected and resuspended in binding buffer (10 mM Hepes/NaOH, 140 mM NaCl and 2.5 mM CaCl_2_). Annexin V-FITC and PI were added, and the reaction mixture was incubated in the dark for 15 min. Cells were analysed by flow cytometry using the FACSCalibur™ flow cytometer.

### Adipogenic and osteogenic differentiation

Adipogenic differentiation was induced by culturing 90% confluent cultures in DMEM/F12 supplemented with 10% FBS, 0.5 mM IBMX (isobutylmethylxanthine), 10 mg/ml insulin, 1 mM dexamethasone and 100 mM indomethacin for 3 weeks. The medium was changed every third day [13].

Osteogenic differentiation was assessed by incubating the cells with DMEM/F12 and 10% FBS supplemented with 0.1 μM dexamethasone, 10 μM β-glycerophosphate and 50 μM ascorbate for 3 weeks. To assess mineralization, cultures were stained with Alizarin Red S (all from Sigma) [14].

### Neuronal differentiation under chemical induction

The *in vitro* neuroectodermal differentiation of pMSCs was evaluated in three replicates by culturing cells for 3–5 passages. At 20% confluence, the pMSCs were induced with neuronal induction media consisting of DMEM/F12, 2% FBS, 20% B27 supplement (Gibco), 20 ng/ml bFGF (basic fibroblast growth factor) and 20 ng/ml EGF. The medium was changed every other day [15].

After 7 days in this medium, cells were transferred into 24-well plates with one coverslip under serum-free conditions in the same medium. In addition to bFGF and EGF, 5 μM RA (Sigma) was added to the medium. The endpoints for induced pMSCs after 7 days of induction have been shown to correspond to the partially differentiated neuronal phenotype [16].

### Immunocytochemistry staining

For immunocytochemistry methods, cells grown in Petri dishes were fixed with 4% PFA in PBS (pH 7.4) for 30 min at room temperature (20–25°C), washed in PBS, treated with 0.3% Triton X-100 in PBS for 10 min and blocked with 3% BSA in PBS for 10 min. The primary antibodies were then added. The cells were incubated with the following primary antibodies for 24 h at 4°C: 1:100 goat anti-Oct4, 1:100 rabbit anti-Nanog (both from Santa Cruz Biotechnology), 1:100 mouse anti-β-tubulin III (Tuj1, Chemicon International), 1:500 rabbit anti-MAP2 (microtubule-associated protein 2; Chemicon International) and 1:400 mouse anti-nestin (BD Biosciences). Cells incubated with PBS without primary antibodies were used as negative controls for marker staining. Subsequently, the cells incubated with the primary antibodies were washed with PBS and incubated with the corresponding secondary antibodies for 30 min at 37°C. Secondary anti-mouse/goat/rabbit antibodies conjugated to Alexa Fluor® 488 or 594 were obtained from Santa Cruz Biotechnology. Immunocytochemistry staining procedures followed a previously described protocol [17]. Nuclei were stained with DAPI (4′,6-diamidino-2-phenylindole) for cell counting.

All experiments were performed in triplicate. The percentage of positive cells was randomly calculated. To perform quantitative analysis, the number of positive cells was counted on each acquired image by ImageJ1.42 (National Institutes of Health). The ratio of positive cells to the number of nuclei was analysed for each antigen.

### RT–PCR (reverse transcription–PCR) and qRT-PCR

Total RNA was extracted from the pMSCs using the RNeasy kit (Qiagen). Thirty PCR cycles were performed. The products were resolved by agarose gel electrophoresis. The primer sequences were designed based on the pig genome, except for Tuj1. We designed the Tuj1 primer based on these conserved sequences from different species. The PCR primers are listed in Table 1.

**Table 1:** RT–PCR primers

Gene	Forward/reverse	Primer	Size (bp)	Annealing temperature (°C)
OCT4	Forward	5′-GTCGCCAGAAGGGCAAAC-3′	157	55
	Reverse	5′-CAGGGTGGTGAAGTGAGGG-3′		
NANOG	Forward	5′-AGCGAATCTTCACCAATGCC-3′	230	53.6
	Reverse	5′-TGCTTCTTGACTGGGACCTT-3′		
Nestin	Forward	5′-GGTGATAGAGCCCGTGTTGG-3′	201	60
	Reverse	5′-TCTTCTCCCAGGGGTGACTC-3′		
MAP2	Forward	5′-GGGATTAGCAGTAACCCACG-3′	224	60
	Reverse	5′-AGGCCATCTGTCCAAAGTCA-3′		
Tuj1	Forward	5′-TCCAGGAGCTGTTCAAGCG-3′	142	61
	Reverse	5′-TCGGACACCAGGTCGTTC-3′		
Osteocalcin	Forward	5′-TCAACCCCGACTGCGACGAG-3′	204	60
	Reverse	5′-TTGGAGCAGCTGGGATGATGG-3′		
PPARγ	Forward	5′-ATTCCCGAGAGCTGATCCAA-3′	203	59
	Reverse	5′-TGGAACCCCGAGGCTTTAT-3′		
GAPDH	Forward	5′-GTCGGAGTGAACGGATTTG-3′	175	53.8
	Reverse	5′-TCTCAGCCTTGACTGTGCC-3′		

The qRT-PCR contained SYBR *Premix Ex Taq*™ (Perfect Real Time, TaKaRa), 0.25 μM of each primer (see Table 1) and cDNA (corresponding to 25 ng of total RNA). qRT-PCR was performed in a 7500 Real-Time PCR system (Applied Biosystems). The level of the test genes were compared with the housekeeping gene GAPDH (glyceraldehyde-3-phosphate dehydrogenase). The results are presented as relative gene expression compared with GAPDH using the 2−ΔΔ*C*_t_ method [18].

### Statistical analysis

Multiple samples were collected for each measurement, and the results were expressed as the means±S.D. Student's *t* test was used to analyse the differences between groups, with differences considered to be statistically significant when *P*<0.05.

## RESULTS

### Morphological characterization of pMSCs

**Figure 1:**
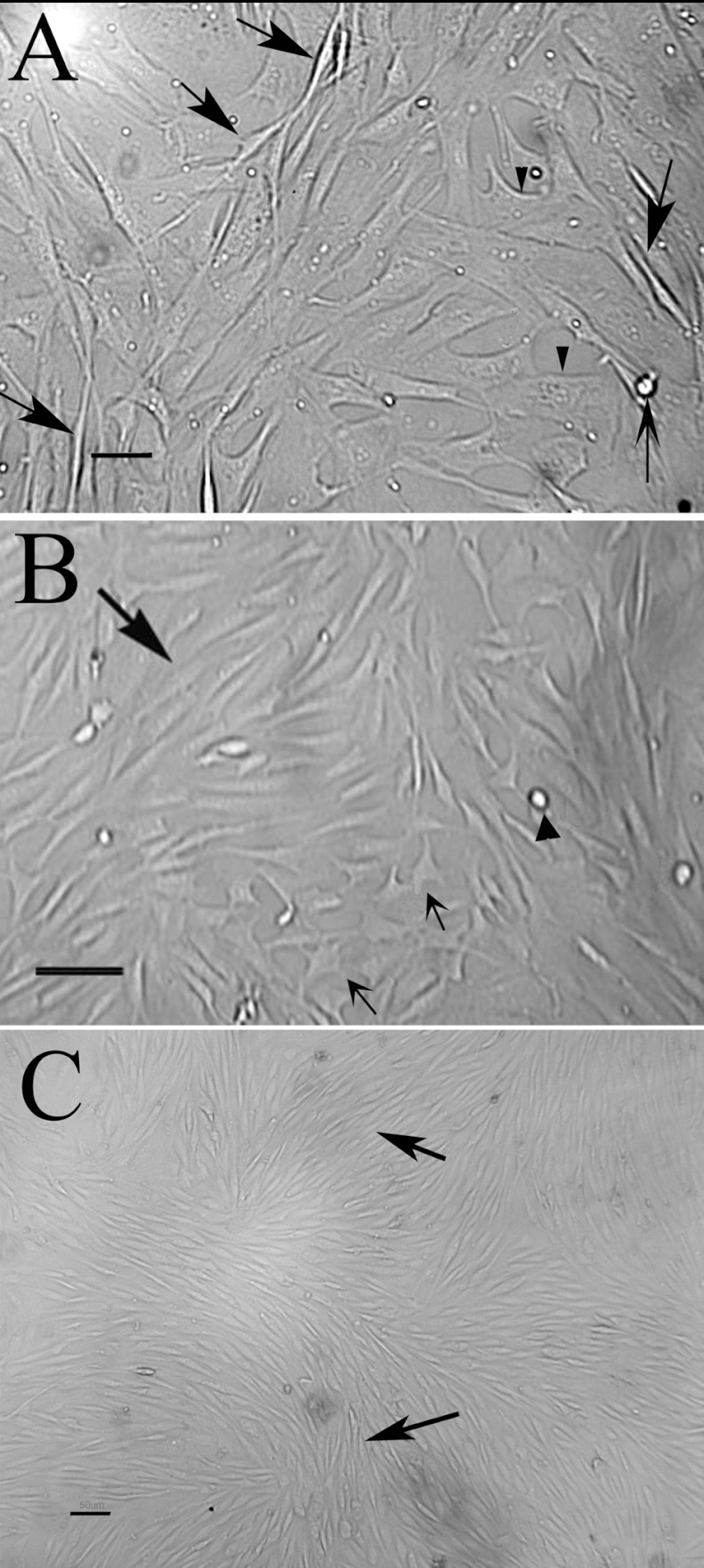
Phase-contrast micrograph of pMSCs in culture (**A**) Passage 1: spindle-shaped cells (arrow), flattened cells (arrowhead); (**B**) Passage 1: spindle-shaped cells (big arrow), star-shaped cells (small arrow) and round cells (arrowhead); (**C**) Passage 3: spindle-shaped cells (arrow). Scale bar: 50 μm.

The pMSCs could be easily isolated from bone marrow based on their adherence to the Petri dish. After plating for 24 h, some pMSCs were adherent to the surface of the Petri dish. The cells at P1 (passage 1) to P2 (passage 2) were heterogeneous and composed of four types: spindle-shaped cells, star-shaped cells, round cells and flattened cells (Figures 1A and 1B). After 3–4 passages, two distinct populations were present when cell numbers were lower in the pMSC cultures: spindle-shaped cells and flattened cells. When the cells were confluent, the majority of cells were spindle-shaped (Figure 1C).

### Expression of surface markers of pMSCs

**Figure 2:**
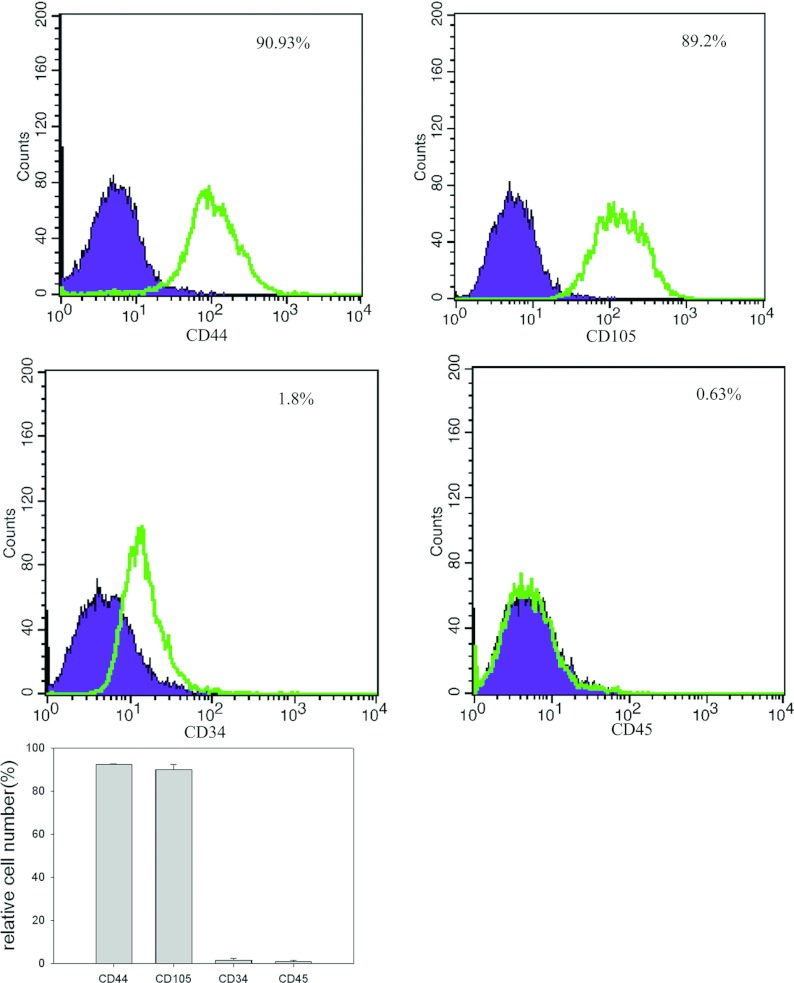
Flow cytometry for surface markers of pMSCs The pMSC suspension was immunostained for CD44, CD105, CD34 and CD45. Cell surface analysis of pMSCs by FACS revealed that pMSCs were positive for CD44 and CD105 but negative for CD34 and CD45. Purple indicates the immunoglobin isotype control. Green indicates specific antibody staining profiles. Data are expressed as the means±S.D. The data represent three individual experiments.

Surface antigens of P3 were analysed using flow cytometry. The results revealed that the pMSCs were positive for CD44 and CD105 and were negative for CD34 and CD45 (Figure 2).

### Proliferation and apoptosis assay of pMSCs

**Figure 3:**
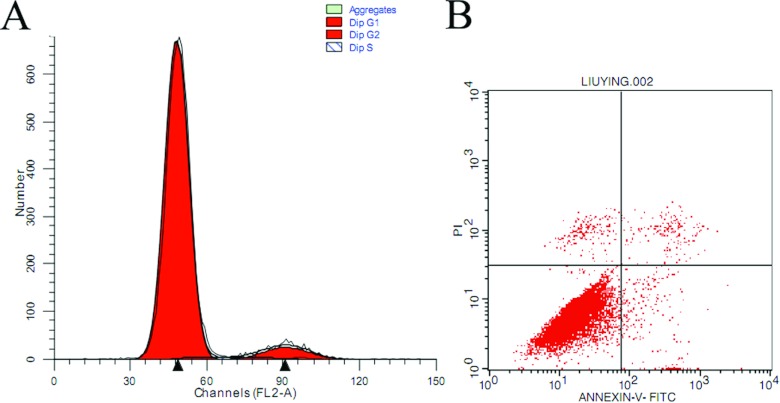
The cell cycle and apoptosis of pMSCs at P10 by flow cytometry (**A**) Cell cycle of pMSCs; pMSCs were under proliferative status. (**B**) The pMSCs were tested for viability with Annexin V and PI staining.

Proliferation is an important characteristic of pMSCs. We analysed the cell cycle of P10 cells using flow cytometry. The results revealed that the pMSCs were under proliferative conditions (Figure 3A). Cell apoptosis was assessed using a standard flow cytometry-based Annexin V/PI apoptosis assay. In this assay, the cells undergoing apoptosis are Annexin V-positive and PI-negative; PI staining designates dead cells, and conversely, a lack of Annexin V staining and PI uptake is observed for live cells. The data showed that cells at P10 maintained viability (Figure 3B).

### Expression of ESC markers in pMSCs and PBs

**Figure 4:**
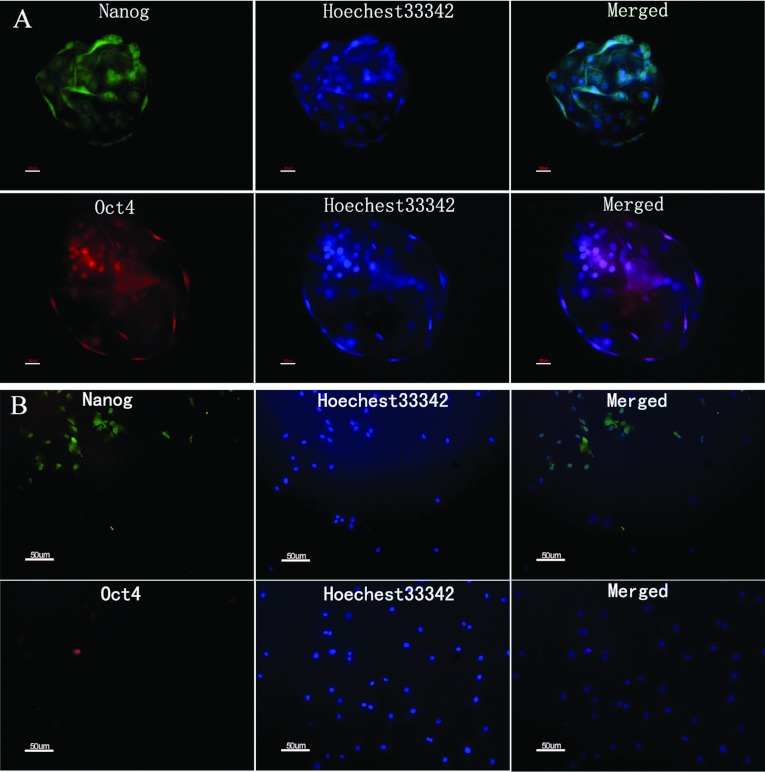
Expression of Oct4 and Nanog in pMSCs and PBs by immunocytochemistry staining (**A**) PBs. (**B**) pMSCs. Oct4 and Nanog were expressed in the nuclei of PBs and pMSCs. Nuclei were stained by Hoechst 33342 (blue). Scale bar: 50 μm.

The expression levels of Oct4 and Nanog were maintained in the ICM (inner cell mass) and the trophoblast of the PB (Figure 4A). The pMSCs at P5 (passage 5) expressed positive immunoreactivity for Oct4 and Nanog (Figure 4B). The Oct4 and Nanog proteins localized to the nuclei in the PBs and the pMSCs.

**Figure 5:**
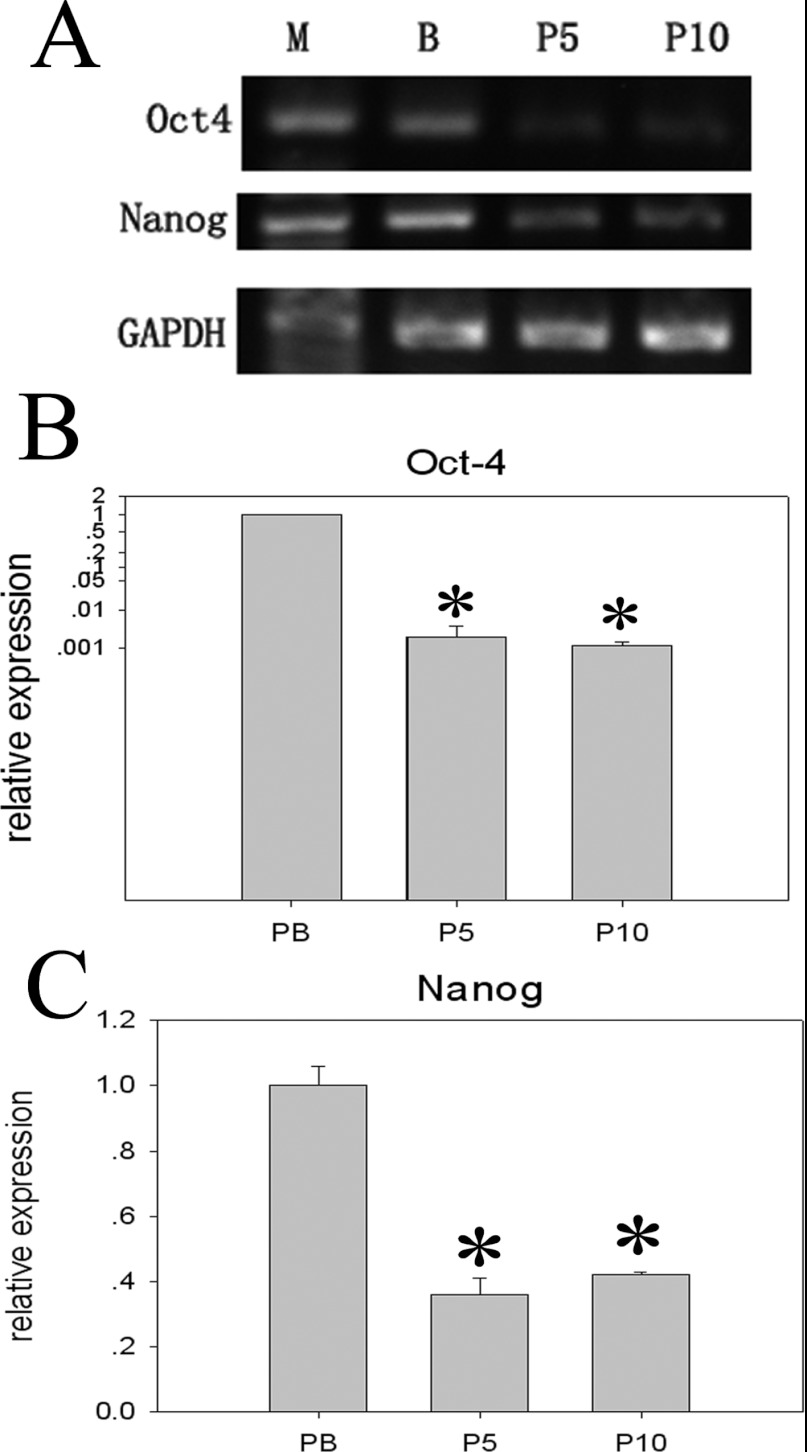
Oct4 and Nanog mRNA expression in the pMSCs at P5 and P10 compared with PBs (**A**) RT–PCR results confirmed expression of Oct4 and Nanog. Expression of GAPDH was used as a loading control. (**B** and **C**) Oct4 and Nanog mRNA were analysed by real-time PCR. Gene expression levels were normalized to GAPDH mRNA, and relative quantification was performed using the ΔΔ*C*_t_ method. The levels of mRNA in the PBs were chosen as a reference and were arbitrarily normalized to 1. Data are expressed as the means±S.D. of triplicates. (*PBs compared with P5 and P10, respectively, *P*<0.05). RT–PCR (**A**): M, DL500 DNA Marker.

RT–PCR and qRT-PCR data showed that the pMSCs expressed the pluripotent markers of Oct4 and Nanog and that the expression levels of Oct4 and Nanog in P5 and P10 cells were significantly lower than those of the PBs (*P*<0.05) (Figure 5). No apparent differences were observed in these markers between P10 and P5 cells (Figure 5).

### Adipogenic and osteogenic differentiation

**Figure 6:**
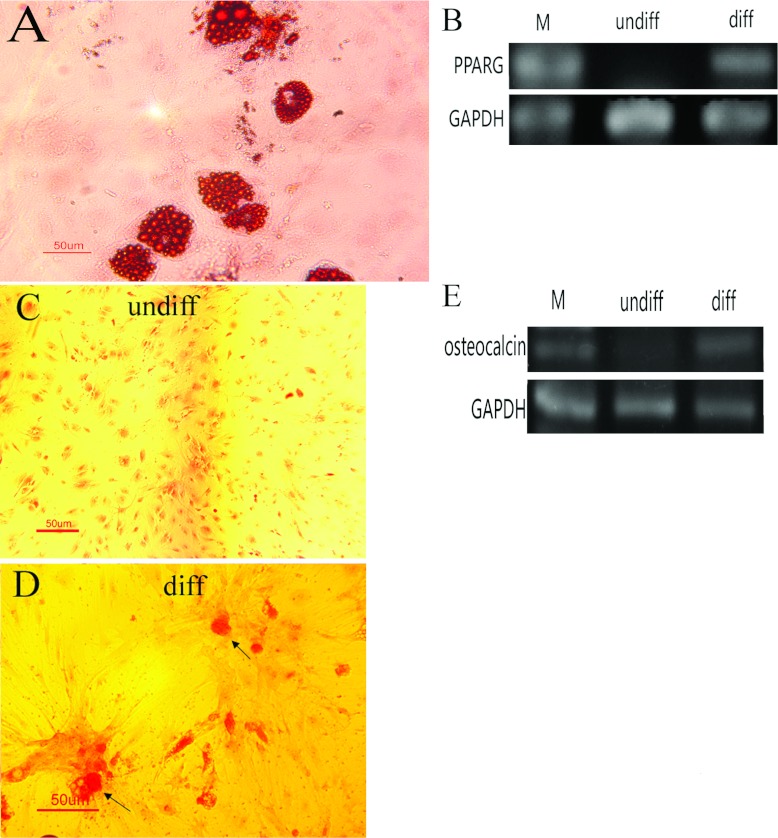
Induced adipogenic and osteogenic differentiation of pMSCs (**A**) The adipogenic lineage differentiation is shown by bright field pictures of positive Oil Red O-stained lipid vacuoles within the cytoplasm of the pMSCs after 21 days of induced differentiation. Scale bar: 50 μm. (**B**) PPARG expression, as shown by RT–PCR in adipogenic-differentiated and undifferentiated pMSC cultures. Osteogenic differentiation after 21 days of treatment with osteogenic medium. Staining with Alizarin Red S (**D**) demonstrates calcium deposits (arrow) typical of osteogenic tissue types when induced compared with non-induced controls (**C**). Scale bar: 50 μm. (**E**) Osteocalcin expression, as shown by RT–PCR in osteogenic differentiated and undifferentiated pMSC cultures. GAPDH was used as an internal control for each PCR. RT–PCR (**B**, **E**): M, DL500 DNA Marker; undiff, non-induced pMSCs; diff, induced pMSCs.

To analyse the differentiation potential of the pMSCs, adipogenic and osteogenic differentiation was induced. pMSCs were cultured in different induction medium. The pMSCs showed adipogenic differentiation (lipid vacuoles stained with Oil Red O) after treatment for 21 days with adipogenic medium (Figure 6A). After treatment for 21 days with osteogenic medium, the pMSCs were embedded in mineralizing matrix containing calcium. The mineralization of the extracellular matrix were assessed by Alizarin Red S staining (Figure 6D). pMSCs in osteogenic medium exhibited a few scattered red-stained calcified nodules. Cells in growth medium did not form calcified nodules (Figure 6C). In accordance with these changes in morphology, the expression of genes associated with adipogenic and osteogenic differentiation, PPARG (peroxisome-proliferator-activated receptor γ) and osteocalcin increased by RT–PCR (Figures 6B and 6E).

### Neuronal differentiation of pMSCs under RA induction

**Figure 7:**
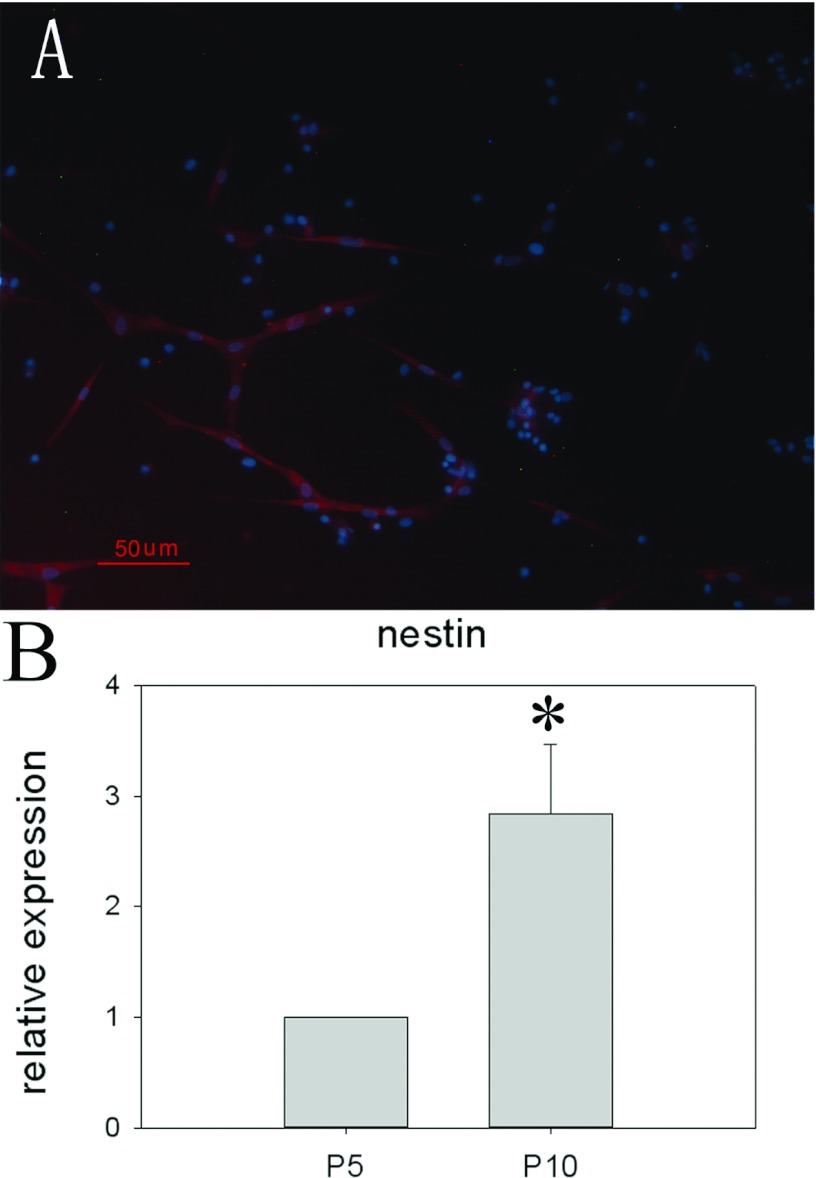
Nestin expression in pMSCs (**A**) Immunofluorescent labelling showed that nestin (red) was expressed by the undifferentiated pMSCs in P5. Scale bar: 50 μm. (**B**) Nestin mRNA was analysed by real-time PCR. Gene expression levels were normalized to GAPDH mRNA, and relative quantification was performed using the ΔΔ*C*_t_ method. The levels of mRNA in P5 were chosen as the reference and were arbitrarily normalized to 1. Data are expressed as the means±S.D. of triplicates.*P5 compared with P10, *P*<0.05.

The pMSCs at P5 were positive for nestin by immunocytochemistry (Figure 7A). The qRT-PCR data showed that nestin expression at P10 was higher than that at P5 (*P*<0.05) (Figure 7B). The pMSCs were differentiated towards the neuronal lineage based on their cellular and molecular marker expression. The pMSCs were then cultured in media containing bFGF, EGF and B27 for 7 days.

**Figure 8:**
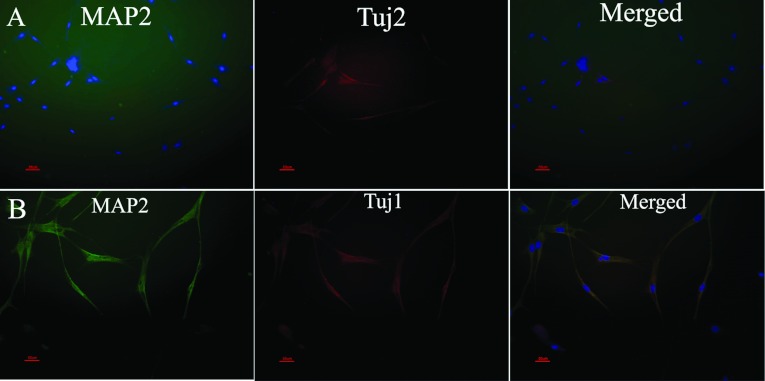
Immunofluorescent labelling showing Tuj1 and MAP2 expression (**A**) Tuj1 (red) was expressed by approximately 4% of the pMSCs; pMSCs were negative for MAP2 (green). (**B**) The neural-like cells derived from the pMSCs were positive for Tuj1 (red, 24%) and MAP2 (green, 29%). Scale bar: 50 μm. Data are expressed as the means±S.D. of triplicates.

**Figure 9:**
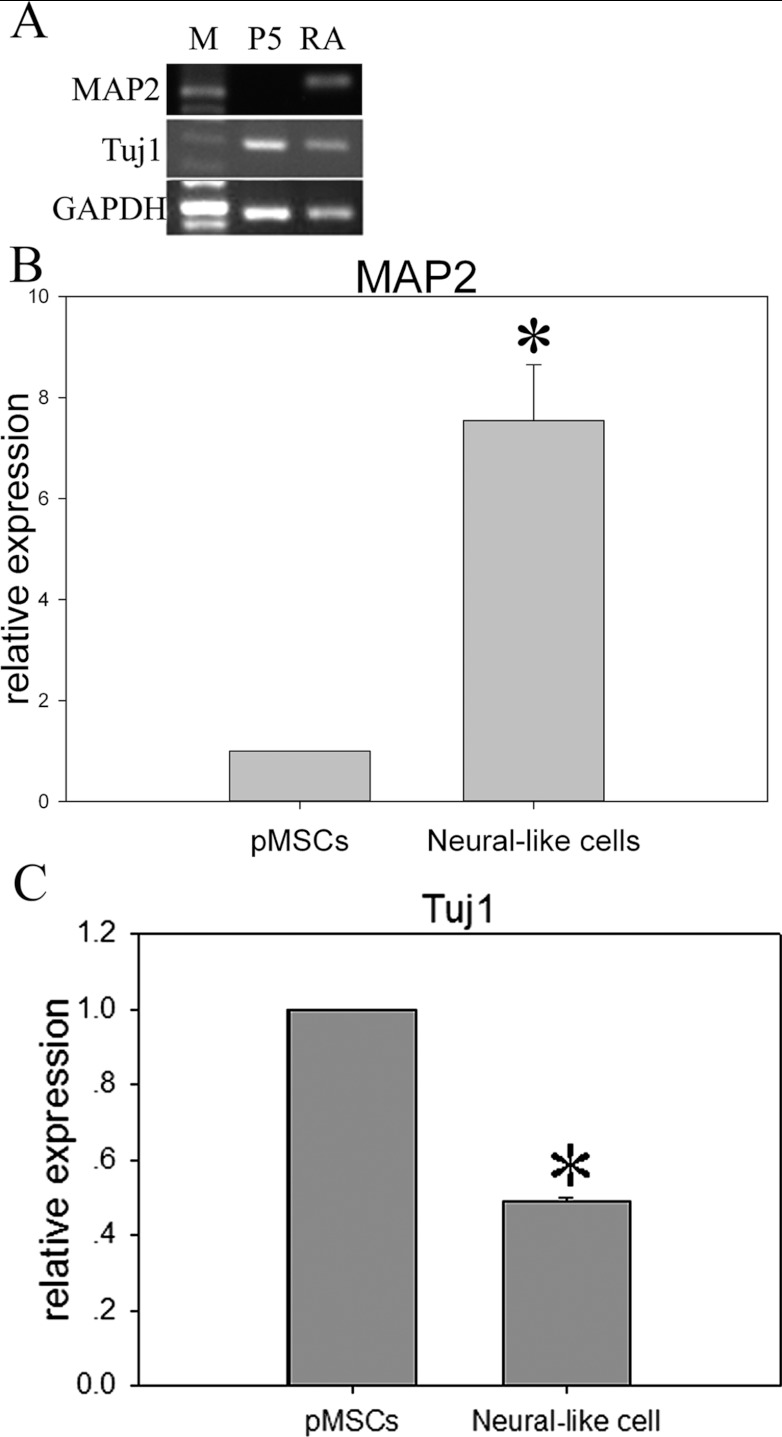
MAP2 and Tuj1 mRNA expression in pMSCs and the neural-like cells derived from pMSCs were analysed by RT–PCR and real-time PCR (**A**) RT–PCR. Expression of GAPDH was used as a loading control. (**B** and **C**) qRT-PCR. Gene expression levels were normalized to GAPDH mRNA, and relative quantification was performed using the ΔΔ*C*_t_ method. The levels of mRNA in the pMSCs were chosen as the reference and were arbitrarily normalized to 1. Data are expressed as the means±S.D. of triplicates. RT–PCR (**A**): M, DL500 DNA Marker; RA, pMSCs with RA added for 7 days. *pMSCs compared with neural-like cells, *P*<0.05.

After 7 days culture in the differentiation medium, the medium was supplemented with RA, and the cells were cultured for an additional 7 days. The pMSCs became thinner and had long protrusions after induction. The pMSCs and neural-like cells derived from the pMSCs were stained by the Tuj1 and MAP2 antibodies. Four per cent of the pMSCs were positive for Tuj1 (Figure 8A). The pMSCs were negative for the mature neuronal marker MAP2 (Figure 8A). After induction, 23.9±10.84% of the pMSC-derived neural-like cells were positive for Tuj1 (Figure 8B), and 29.65±13.09% of the pMSC-derived neural-like cells were positive for MAP2 (Figure 8B). The immunocytochemistry analysis was confirmed by the RT–PCR and qRT-PCR data for the neural markers MAP2 and Tuj1 at the RNA level (Figure 9), but the Tuj1 mRNA level was far below the protein level (Figure 9).

## DISCUSSION

MSCs are an interesting model cell type for the investigation of differentiation mechanisms due to the relative ease of establishment of *in vitro* cultures and their good proliferation ability [19]. Multipotent MSCs are presently the most promising cell source for therapeutic applications. Many traits of porcine animal models make them desirable for the study of new therapeutic approaches for human diseases. Pigs also have many well-characterized homologues for human genetic diseases, making them an ideal large animal model in which to evaluate gene-therapy protocols [20]. Recent findings about the lack of a significant host immune response to the xenograft of porcine umbilical cord matrix cells, suggests that the transplantation of porcine cells may provide a therapeutic approach for the treatment of neurodegenerative diseases [21]. The aim of this study was to evaluate the growth and differentiation characteristics of pMSCs.

In this study, pMSCs were successfully isolated utilizing the property of adherence to plastic, and following culture, the early cell population showed spindle, flattened, star and round morphologies. When the cells become confluent, round cells that float above the substrate begin appearing. Round cells can be removed by replacing the culture medium. Although the adherent cells varied in size and phenotype, the most abundant cells noticeable were the spindle-shaped cells. The spindle-shaped cells formed a larger colonies compared with the other three types of cells, which were more dispersed. More recent studies indicate that single-cell-derived colonies are morphologically heterogeneous, containing at least two different cell types: spindle-shaped cells and flattened cells [22,23]. The flattened cells replicate slowly and appear more mature [24–26]. The proportion of spindle-shaped cells remains high for several passages if the cultures are maintained at low density, but the flattened cells were predominant in later passages when the cells cease proliferating [25–28]. Furthermore, these cells displayed the characteristic surface antigen expression pattern of culture-expanded pMSCs because they were positive for well-defined markers such as CD44 and CD105. In accordance with the reported phenotype of pMSCs, the expression of CD44 in the pMSCs indicates the level of cell–cell and cell–matrix interactions and the potential role of these interactions in supporting homing mechanisms at residing or target tissues [29,30]. CD44 has been recently proposed to be involved in stem cell pluripotency and marks several types of cancer stem cells [31]. CD105, also known as endoglin, is the TGFβ (transforming growth factor β) receptor III, which potentially plays a role in TGFβ signalling during MSC osteogenic differentiation [32]. In addition, the haematopoietic cell markers CD45 and CD34 were not detected in the pMSCs, suggesting that the pMSCs did not come from the haematopoietic cell derivative. Based on the expression of a panel of surface markers, our results support the identity of isolated cells as MSCs.

Adherent cells from porcine bone marrow were found to actively proliferate *in vitro* and to maintain their morphological and growth characteristics for over 10 passages. Previous reports support an unexpected similarity between human MSCs and ESCs [32–38]. In our study, cells grown in standard media showed spindle-shaped and flattened cells and had the ability to continuously divide, as has been observed for bone marrow stroma and tissue-specific MSCs from other species [39,40].

Following adipogenic and osteogenic induction, the pMSCs in this study exhibited cytoplasmic lipid droplets and an accumulation of calcium nodules, respectively. Several reports have shown the ability of pMSCs isolated from different tissue sources to differentiate *in vitro* into fat and bone cells [41–43].

We found that pMSCs also expressed the ESC markers Oct4 and Nanog using immunocytochemistry and PCR. The expression of pluripotent markers is indicative of cells that have the capacity to differentiate into cell types derived from all three germ layers. The transcription factors Oct4 and Nanog are expressed at high levels in ESCs [44], and their expression in ESCs indicates the maintenance of undifferentiated pluripotency [45,46]. Our study also showed that these transcription factors were expressed in both blastocysts and the pMSCs (Figure 4).

The expression of Oct4 and Nanog in pMSCs may explain the ability of pMSCs to differentiate into neural-like cells. This study not only assesses the stemness of bone marrow-derived cells but also investigates the ability of these cells to differentiate into neurons. Moreover, pMSCs can directionally differentiate into neurogenic cells, as demonstrated by their levels of RNA and protein expression. Nestin is widely considered to be a specific marker of NSCs (neural stem cells) and progenitors [46,47]. Under standard culture conditions, non-induced pMSCs did not express MAP2, but a small fraction of cells were positive for nestin [46]. The fact that the expression of nestin was higher in P10 cells than in P5 cells may indicate that the pMSCs naturally differentiate. The presence of nestin-positive cells in the untreated pMSCs suggests that these cells carry true neuronal potential that is ready to be activated under the appropriate conditions. Our results showed that the normal pMSCs also expressed the neural marker β-tubulin III, suggesting that the pMSCs were ‘multidifferentiated’ cells and thus could thus retain the ability for neuronal differentiation, enhancing their ability to be employed in the treatment of neurological diseases. A previous report [48] showed that, after several passages, MSCs expressed neuronal genes and proteins, as demonstrated by RT–PCR and Western blotting. Our results showed that pMSCs could be induced to differentiate into neural-like cells. The expression level of the mature neuronal marker MAP2 increased obviously in pMSCs at both the protein and molecular levels after induction. The mRNA level of the immature neuronal marker Tuj1 significantly decreased in pMSCs after induction.

In summary, successfully isolated pMSCs displayed the typical morphology and surface antigen profile, and had the ability to differentiate into osteocytes and adipocytes. Furthermore, distinctive morphological characteristics and the expression of markers specific to neuronal phenotypes supported their potential to differentiate *in vitro* into neural-like cells upon exposure to appropriate stimuli. The present study improves our knowledge about the plasticity of pMSCs and facilitates further studies dedicated to the search for the best source of MSCs for cellular replacement therapy for numerous diseases and trauma states.
